# Brain structural alterations in vestibular schwannoma beyond tinnitus and hearing loss

**DOI:** 10.1093/braincomms/fcaf107

**Published:** 2025-03-11

**Authors:** Abraham A Adegboro, Ziyan Chen, Jens J Peters, Cyrille D Dantio, Siyi Wanggou, Chubei Teng, Xuejun Li

**Affiliations:** Department of Neurosurgery, Xiangya Hospital, Central South University, Changsha, Hunan 410008, PR China; National Clinical Research Center for Geriatric Disorders, Xiangya Hospital, Central South University, Changsha, Hunan 41008, PR China; Hunan International Scientific and Technological Cooperation Base of Brain Tumour Research, Xiangya Hospital, Central South University, Changsha, Hunan 410008, PR China; Department of Neurosurgery, Xiangya Hospital, Central South University, Changsha, Hunan 410008, PR China; National Clinical Research Center for Geriatric Disorders, Xiangya Hospital, Central South University, Changsha, Hunan 41008, PR China; Hunan International Scientific and Technological Cooperation Base of Brain Tumour Research, Xiangya Hospital, Central South University, Changsha, Hunan 410008, PR China; Department of Neurosurgery, Xiangya Hospital, Central South University, Changsha, Hunan 410008, PR China; National Clinical Research Center for Geriatric Disorders, Xiangya Hospital, Central South University, Changsha, Hunan 41008, PR China; Hunan International Scientific and Technological Cooperation Base of Brain Tumour Research, Xiangya Hospital, Central South University, Changsha, Hunan 410008, PR China; Department of Neurosurgery, Xiangya Hospital, Central South University, Changsha, Hunan 410008, PR China; National Clinical Research Center for Geriatric Disorders, Xiangya Hospital, Central South University, Changsha, Hunan 41008, PR China; Hunan International Scientific and Technological Cooperation Base of Brain Tumour Research, Xiangya Hospital, Central South University, Changsha, Hunan 410008, PR China; Department of Neurosurgery, Xiangya Hospital, Central South University, Changsha, Hunan 410008, PR China; National Clinical Research Center for Geriatric Disorders, Xiangya Hospital, Central South University, Changsha, Hunan 41008, PR China; Hunan International Scientific and Technological Cooperation Base of Brain Tumour Research, Xiangya Hospital, Central South University, Changsha, Hunan 410008, PR China; Department of Neurosurgery, Xiangya Hospital, Central South University, Changsha, Hunan 410008, PR China; National Clinical Research Center for Geriatric Disorders, Xiangya Hospital, Central South University, Changsha, Hunan 41008, PR China; Hunan International Scientific and Technological Cooperation Base of Brain Tumour Research, Xiangya Hospital, Central South University, Changsha, Hunan 410008, PR China; Department of Neurosurgery, Xiangya Hospital, Central South University, Changsha, Hunan 410008, PR China; National Clinical Research Center for Geriatric Disorders, Xiangya Hospital, Central South University, Changsha, Hunan 41008, PR China; Hunan International Scientific and Technological Cooperation Base of Brain Tumour Research, Xiangya Hospital, Central South University, Changsha, Hunan 410008, PR China; Xiangya School of Medicine, Central South University, Changsha, Hunan 410008, PR China

**Keywords:** vestibular schwannoma, neuroimaging, neuroplasticity, MRI, conservative therapy

## Abstract

Brain tumours alter brain structures and functions. However, morphometric alterations induced by unilateral vestibular schwannoma, a benign tumour of the vestibulocochlear nerve, have not been extensively explored. Recent studies have suggested that the tumour does not grow bigger following diagnosis in several patients, suggesting an avenue for conservative therapy. This study aims to comprehensively investigate brain structural re-organizations in vestibular schwannoma patients taking into account the effects of hearing loss and tinnitus-the most common symptoms. To this end, preoperative data from 48 vestibular schwannoma pathology-confirmed patients and a healthy control group of 30 volunteers were retrospectively included in this study. The clinical and imaging data from these participants were processed. General linear models were designed to identify tumour-related brain alterations in grey matter volume and cortical thickness, alongside three other surface measures: sulcal depth, gyrification index and fractal dimension. The differences obtained were further analysed for correlation with tumour size and pure tone audiometry. Interestingly, grey matter volume, cortical thickness and for the first time, fractal dimension measures were increased in vestibular schwannoma patients across key frontal regions (*P_FWE_* < 0.05). The precuneus, superior and inferior frontal gyrus had increased grey matter volumes and cortical thickening in patients compared to controls, among other changes (*P _FWE_* < 0.05). Meanwhile, the sulcal depth and gyrification index measures demonstrated no significant alterations. Furthermore, grey matter volume changes at the paracentral lobule and precuneus were positively correlated to the tumour size, while the fractal dimension at the superior frontal sulcus was negatively correlated. Finally, grey matter volume increase at the inferior frontal gyrus and cortical thickening at the supramarginal gyrus were negatively correlated to pure tone audiometry. These findings suggest that factors beyond hearing loss and tinnitus contribute to brain structural alterations in this tumour, a better understanding of which might pave the way for non-surgical symptomatic therapies.

## Teaser

Adegboro *et al*. report increased grey matter volume, fractal dimension, and cortical thickness in key frontal regions of vestibular schwannoma patients after adjusting for age, sex, hearing loss and tinnitus. These findings contribute to a more nuanced understanding of the tumour and highlight potential targets for non-invasive management.

## Graphical Abstract

**Graphical Abstract:**
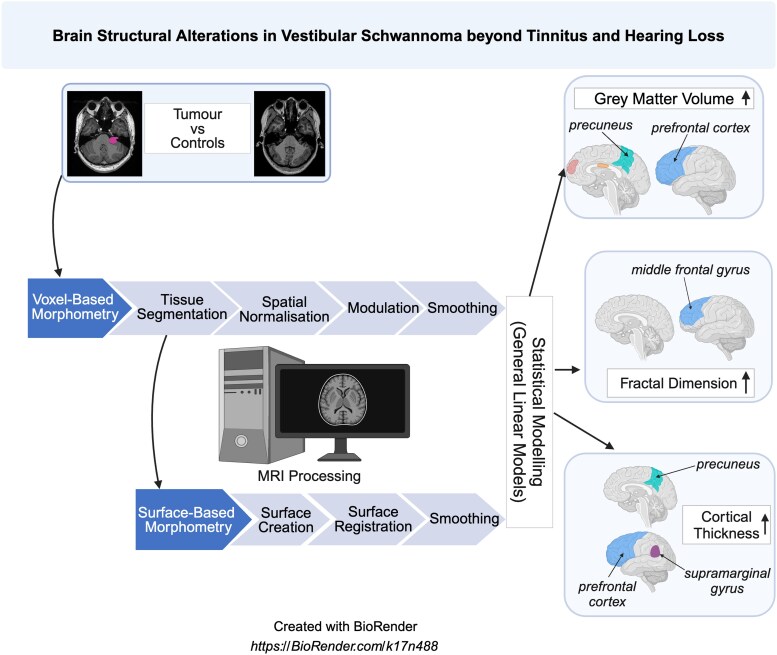

## Introduction

Vestibular schwannoma (VS) is a benign tumour originating from the myelinating Schwann cells arising from the vestibular branch of the vestibulocochlear nerve. They represent about 85% of intracranial cerebellopontine angle (CPA) growths and around 8% of all intracranial tumours.^1^ The lifetime prevalence of VS is estimated to exceed 1 per 500 persons, although this rate might vary across the globe, as different races have shown differences in the rate of incidence.^2^ While rarely life-threatening, it significantly disrupts patients’ quality of life with symptoms such as hearing loss, tinnitus, vertigo, and dizziness, among others. Management options currently include surgery and radiotherapy, yet these can lead to additional complications and, sometimes, do not fully improve the quality of life of patients in situations where the presenting symptom persists post-treatment. Conservative management has been explored, with care centres implementing the ‘watch, wait and rescan’ treatment pathway.^3^ However, symptomatic treatment options for effective conservative management are still considerably limited.

Advanced neuroimaging techniques, such as voxel-based morphometry (VBM) and surface-based morphometry (SBM), have revolutionized our understanding of brain structural changes associated with various neurological conditions. VBM is an automated computational method that examines variations in the regional grey matter (GM) volume by conducting a voxel-wise comparison of the local concentration between two groups.^4^ SBM has demonstrated increased accuracy for brain registration,^5^ making it potentially more sensitive to morphological alterations than VBM. The distance between the GM ribbon’s internal and external border, measured at thousands of points, estimates the GM width, captured as the cortical thickness (CT) in SBM.^6^ SBM additionally measure features such as; the gyrification index (GIx)—the ratio of total cortical surface area (cSA) to outer cSA, sulcal depth (SD)—determined by the Euclidean distance between the central surface and its convex boundary, and fractal dimension (FDi)—a measure of the intricacies of cortical folding patterns, providing a more holistic understanding of cortical complexity and folding. Combining VBM and SBM, in addition to improving the understanding of brain neurobiology significantly improves the detection accuracy of morphological changes.^7^

Recent VS neuroimaging studies comparing VS and controls using VBM and SBM have reported significant GM volume and CT alterations in regions such as the precuneus, middle frontal and temporal gyrus, fusiform gyrus, superior parietal lobule, superior frontal gyrus, etc. showing possible neuroplastic and compensatory mechanisms.^8,9^ However, some of these studies did not consider hearing loss and tinnitus in their models^9^ and only reported CT alterations on the surfaces, which although significant, is only one of several surface measures to be explored in understanding brain morphology, as already exemplified in other pathologies.^10^ Wang and colleagues^11^ reported reductions in GM volume in auditory and visual areas, which are in line with current literature, while the motor system and the somatosensory regions had increased volumes. These non-auditory area changes were attributed to neuroplasticity following hearing loss.^11^ However, this study did not specifically control for other symptoms as well.

This study aims to conduct a comprehensive morphometric analysis of brain structural alterations in VS, implementing the VBM and SBM techniques. Specifically, it will analyse differences in GM volume, CT, GIx, SD and FDi between VS patients and controls to disentangle the neuroplastic changes attributable to the tumour while controlling for hearing loss and tinnitus. It further aims to relate these neuroanatomical changes to tumour size and pure tone audiometry (PTA). Understanding these changes can provide new insights into the neuroplastic mechanisms of VS and inform more holistic management approaches.

## Materials and methods

### Subject recruitment

Pre-operative clinical and radiological data of all pathologically confirmed VS patients from January 2019 to August 2022 from the Neurosurgery Department of Xiangya Hospital, Central South University were collected for this study. In addition, a healthy control group was selected from our previous study. In total, 78 participants were included with 48 VS patients and 30 controls. Informed consent was obtained from the healthy participants, while this requirement was waived for the patient group as data was retrospectively obtained. The Xiangya Hospital, Central South University’s Ethics Review Committee approved this study (No.202407137). To ensure patient privacy, the clinical, imaging and histopathological data were anonymized before processing and analysis and all procedures followed the Declaration of Helsinki.

Inclusion Criteria: (1) Clinical diagnosis and histopathologically confirmed VS; (2) Completed pre-operative non-contrast enhanced 3D T1WI (three-dimensional T1 weighted Imaging) MRI; (3) Availability of MRI sequence on the picture archiving and communication system (PACS) of Xiangya Hospital; and (4) Image quality rating (IQR) after pre-processing, > 70% (described in the MRI pre-processing section).

Exclusion Criteria: (1) Patients with NF2-related schwannomatosis; (2) Incomplete clinical and MRI data; and (3) T1WI MRI sequence with deformation, noise and bad quality (Supplementary Fig. 1).

### MRI Acquisition

Given the retrospective nature of our study, MRI data were acquired using several MRI machines and setups across three centres. Xiangya Hospital data were acquired using Siemens Aera, Siemens Prisma, GE Signa Architect, Ge Signa HDxt, and GE Signa Premier. Feiyu hospital data, available through the Xiangya PACS, were acquired on a GE Discovery MR750w, and some of the healthy control group data were acquired at Xiangya Boai Hospital’s Siemens Skyra. Supplementary Table 1 shows the details of these acquisitions and the different MRI parameters. The acquisition modality employed is the high-resolution magnetization-prepared rapid gradient echo (MPRAGE).

### MRI processing

#### Data format conversion

All collected raw MRI data underwent a uniform format conversion, transforming the original DICOM format into the simpler Neuroimaging Informatics Technology Initiative (NIFTI) format, which is more suitable for subsequent software reading and processing. The ‘dcm2niix’ window’s command line implementation was utilized.^12^ No personal information was processed at this conversion stage to foster data anonymization and protect the participant's identity.

#### Pre-processing

Statistical Parametric Mapping (SPM12, https://www.fil.ion.ucl.ac.uk/spm)^13^ and its Computational Anatomy Toolbox (CAT12, https://neuro-jena.github.io/cat)^6^ toolbox on MATLAB R2021b (The MathWorks Inc., Natick) were utilized for processing all T1-weighted images. The CAT12 automated cross-sectional processing pipeline was employed for these analyses.

The anterior commissure and bicommissural line on the horizontal axis were set as the origin of all T1-weighted images to match the SPM tissue defaults. The CAT12 pre-processing pipeline began with an interpolation step, followed by denoising, which helped to address variability in the MRI raw images. It used the Spatial-adaptive Non-Local Means (SANLM) filter for denoising.^14^ An initial affine pre-processing was performed on this bias-corrected image to improve initial segmentation, followed by a local adaptive segmentation step, which accounted for the variability in GM intensity across brain regions and helped check for variations across different MRI machines. An adaptive maximum a posterior (AMAP) segmentation followed, which helped to further address variability in local intensity. Partial volume segmentation provided an estimation used in segmenting the brain into the various tissue types GM, white matter (WM) and cerebrospinal fluid (CSF); this ensured precise segmentation. Skull-stripping was accomplished using the revised graph-cut technique. Using DARTEL (Diffeomorphic Anatomical Registration Through Exponentiated Lie algebra)^15^ and Geodesic shooting registration,^16^ the segmented images were modulated and normalized to the Montreal Neurological Institute (MNI) standardized space and resampled to a voxel size of 1.5 × 1.5 × 1.5mm^3^. Quality control was the final pre-processing step. It evaluated parameters such as noise, inhomogeneities and image resolution. Image quality measures ranging from 100 rating points to 0, describing excellent to unacceptable image quality respectively, were generated before CAT12 processing. All included images had ratings greater than 70% meeting the satisfactory level typically expected from clinical data.

A Koos grade^17^ was assigned to each tumour (I = entirely intra-meatal, II = both intra- and extra-meatal, III = CPA cistern filled, IV = brainstem compressed), and they were further segmented from each participant's 3D T1W MRI images using the semi-automatic segmentation module provided by ITK-SNAP^18^ (http://www.itksnap.org/) by two experienced neurosurgeons. A third, more experienced neurosurgeon reviewed each segmentation with region of interest overlap of < 90%. Nibabel (https://nipy.org/nibabel/), a Python package, was utilized to load the binary mask, calculate the volume of the delineated segment and estimate the tumour size.

#### Voxel-Based morphometry processing

After the pre-processing above was completed, the GM segment for each participant was obtained. Sample homogeneities were examined to determine and exclude the most deviating images. A Gaussian smoothing kernel of 7 mm full width at half maximum (FWHM) was applied to the extracted GM segment to suppress noise and enhance statistical power.

#### Surface-Based morphometry processing

During the pre-processing phase, CT and central surfaces of the left and right hemispheres were estimated using the projection-based thickness (PBT) technique. Topology correction was employed, followed by spherical mapping. Spherical registration was achieved with the volume-based DARTEL adapted to the surface. Sample homogeneities were examined to view and exclude the most deviating images. The surfaces of the segmented images for CT were smoothed with a Gaussian kernel of 15 mm FWHM to suppress noise and enhance statistical power. Additional surface measures such as the gyrification index (GIx), sulcal depth (SD) and fractal dimension (FDi) were extracted and smoothed with a 20 mm FWHM Gaussian kernel.

#### MRI Harmonization

Since our data originated from multiple sites and various MRI machines, the need to correct for variability introduced by this factor was inevitable. We utilized neuroCombat (https://github.com/Jfortin1/neuroCombat), a Python implementation of combat harmonization^19-21^ to correct scanner variability in the extracted measures and total intracranial volume (TIV) estimated following the pre-processing in CAT12. The various scanners were entered as the confounding batch, while age, sex and disease status (presence or absence of a tumour) were modelled as biological factors of interest.^22^

### Statistical analysis

All imaging statistical analyses were performed using Matlab's CAT12 basic model module. Baseline demographic information for all patients was analysed in Statistical Package for the Social Sciences (SPSS) (IBM SPSS Statistics for Windows, Version 26.0). Unless otherwise stated, *P* < 0.05 was set as the significance threshold for all analyses in this study.

Using SPSS, a two-sample *t*-test was designed to test for differences in age distribution between the VS group and the healthy control (nC) group. Sex differences were tested with Pearson’s chi-square test.

General Linear Models (GLMs) were fitted in CAT12 with *P* < 0.001 set as a priori threshold in the VBM and *P* < 0.005 for the SBM. To correct for multiple comparisons, the Holm-Bonferroni family-wise error (FWE) with a threshold of *P* < 0.05 was considered significant at the cluster level.

A two-sample *t*-test in the CAT12 ‘basic model’ module was fitted to test for GM volume differences in the VBM analysis and CT, GIx, SD and FDi in the SBM analysis to elucidate morphological changes attributable to VS. Age,^23^ sex,^24^ presence or absence of hearing loss and tinnitus were modelled as covariates, as they have been implicated by previous studies to cause brain changes. TIV was modelled as a nuisance variable in the VBM analysis. After modelling, our VBM model was estimated using the classical Restricted Maximum Likelihood (ReML) in SPM12, while CAT12 was used to estimate the SBM model. Contrasts were defined after model estimation, and results were visualized.

The Anatomical Atlas Labelling (AAL3v1)^25^ atlas was used to label the VBM result, while the Destrieux atlas (aparc_a2009s)^26^ was used for labelling the SBM regions. Using the results from the VBM and SBM analysis, Python (3.8.18) library—scipy and Seaborn was employed to calculate and visualize the linear correlation between the altered volume and surface parameters, tumour sizes and PTA. PTA values were estimated as the averages of audiometric values at 500, 1000, 2000 and 4000db HL,^27^ and the PTA correlations were assessed only on patients with available PTA data.

## Results

### Clinical Characteristics

Following evaluation, 78 participants met the inclusion criteria for this study, comprising 48 VS patients and 30 normal controls (nC). There were 41 (52.6%) males and 37 (47.4%) females with an average age of 43.64 ± 14 years (ranging from 15 to 74). However, the VS groups were older than the controls. Sex distribution between the groups had no statistically significant difference. The WHO hearing impairment grading system^27^ was presented to provide a breakdown of hearing across the VS group using only available PTA values (*n* = 40). Furthermore, a majority of patients (35/48) presented with a Koos grade IV tumour, representing large tumours with significant distortion of the brainstem and cranial nerves. A breakdown of the distribution of other co-morbidities implicated in brain reorganizations was also provided (Table 1).

**Table 1:** Demographic information of study participants

	VS, *n* = 48	Controls, n = 30	Statistics	*P*-Value
Age (years)	48 ± 14 [15, 74]	36 ± 9 [21, 58]	6.09	0.02*
Sex, Male/Female	24/24	17/13	0.33	0.57
Tinnitus, Present/Absent	25/23	n/a		
Hearing loss, Yes/No	36/12	n/a		
Headache, Yes/No	8/40	n/a		
Dizziness, Yes/No	15/33	n/a		
Tumour side, Left/Right	24/24	n/a		
Tumour size (mm^3^)	5023 (203, 62018)	n/a		
**Koos Grading Scale**				
I	0			
II	6			
III	7			
IV	35			
PTA (db HL)	48 ± 30 [15, 117]	n/a		
**WHO, Hearing Impairment Grading System**				
Grade 0 (PTA < 20db HL)	3/40			
Grade 1 (20–34db HL)	3/40			
Grade 2 (35–49 db HL)	8/40			
Grade 3 (50–64 db HL)	8/40			
Grade 4 (65–79 db HL)	12/40			
Grade 5 (PTA > 80 db HL)	6/40			
**Comorbidities**				
Hypertension, Yes/No	9/39	n/a		
Type II Diabetes, Yes/No	3/45	n/a		
Smoking history, Yes/No	5/43	n/a		

Age and PTA are presented as Mean ± standard deviation (min, max), and age was analysed by *t*-test. Categorical variables are presented as ratios, and sex was analysed by Pearson’s chi-square test. Tumour size was presented as median (min, max). The WHO hearing impairment grading system classifies hearing as no impairment (Grade 0), mild (Grade 1), moderate (Grade 2), moderately severe (Grade 3), severe (Grade 4) and profound impairment (Grade 5). WHO = World Health Organization, VS = vestibular schwannoma, PTA = pure tone audiometry, n/a = not applicable, db HL = decibels Hearing Level.

^*^Statistical significance.

### Vestibular schwannoma-related neuroplastic changes

This section provides an overview of the main morphological changes attributable to the tumour. Following the orthogonality check for these models, hearing loss and tinnitus had a significant correlation (*r* = 0.63), as reported by the SPM module. To ensure model stability and reliability, we modelled both as covariates alongside age and sex with interaction effects to disentangle the effects of each and control for multicollinearity issues.

#### Tumour voxel-based morphometry

**Figure 1:**
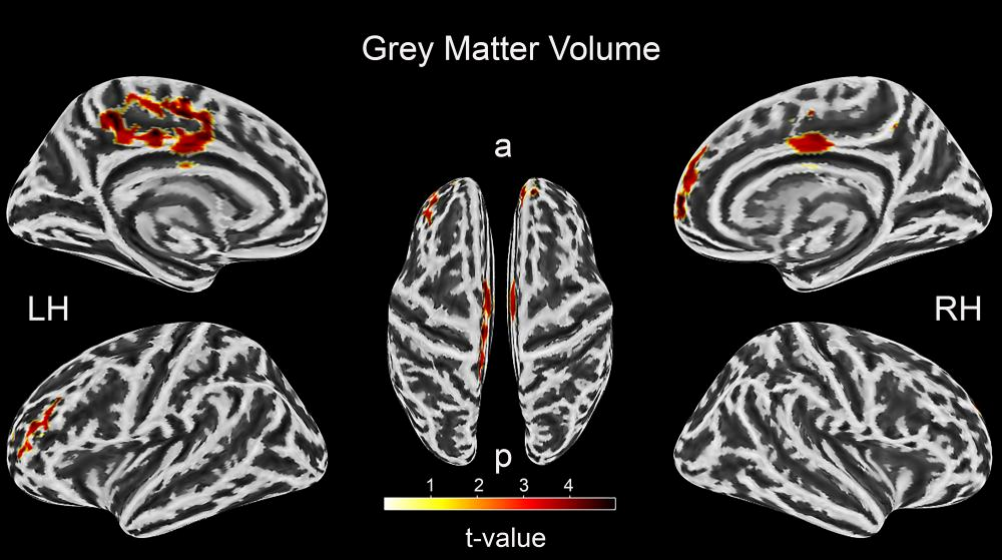
**Tumour VBM changes.** Two-sample *t*-test; *P* < 0.05, FWE corrected, *N* = 78. The figure illustrates the *t*-values of clusters where the tumour group had higher grey matter volume than the normal control group, with strength indicated by varying intensity across regions. a = anterior, *P* = posterior, LH = left hemisphere, RH = right hemisphere.

The VBM result showed that the VS group, relative to the nC group, had increased GM volume (with Cohen’s effect size (*d*) ranging from *d* = 0.78 to *d* = 1.43) in the bilateral middle cingulate and paracingulate gyri (MCC), superior frontal gyrus-medial, left supplementary motor area (SMA), left paracentral lobule (PCL), left precuneus, right superior frontal gyrus—dorsolateral—medial orbital, left middle frontal gyrus (MFG), left inferior frontal gyrus—triangular part and left superior frontal gyrus—dorsolateral (*P_FWE_* < 0.05 at the cluster level; Table 2 and Fig. 1).

**Table 2:** Tumour changes VBM results. VS > nC (*P* < 0.05, FWE corrected)

*P*-Value	*t*-Value	Cluster size	MNI coordinates (mm)			Anatomical regions
			*x*	*y*	*Z*	
Grey matter volume						
1.30E-06	5.90	2071	9	−12	52	Left middle cingulate & paracingulate gyri
						Right middle cingulate & paracingulate gyri
						Left supplementary motor area
						Left paracentral lobule
						Left precuneus
1.40E-05	4.90	973	−6	60	4	Right superior frontal gyrus-medial
						Right superior frontal gyrus-dorsolateral
						Left superior frontal gyrus-medial
						Right superior frontal gyrus-medial orbital
7.70E-05	4.10	574	39	38	22	Left middle frontal gyrus
						Left inferior frontal gyrus-triangular part
						Left superior frontal gyrus-dorsolateral

#### Tumour surface-based morphometry

**Figure 2:**
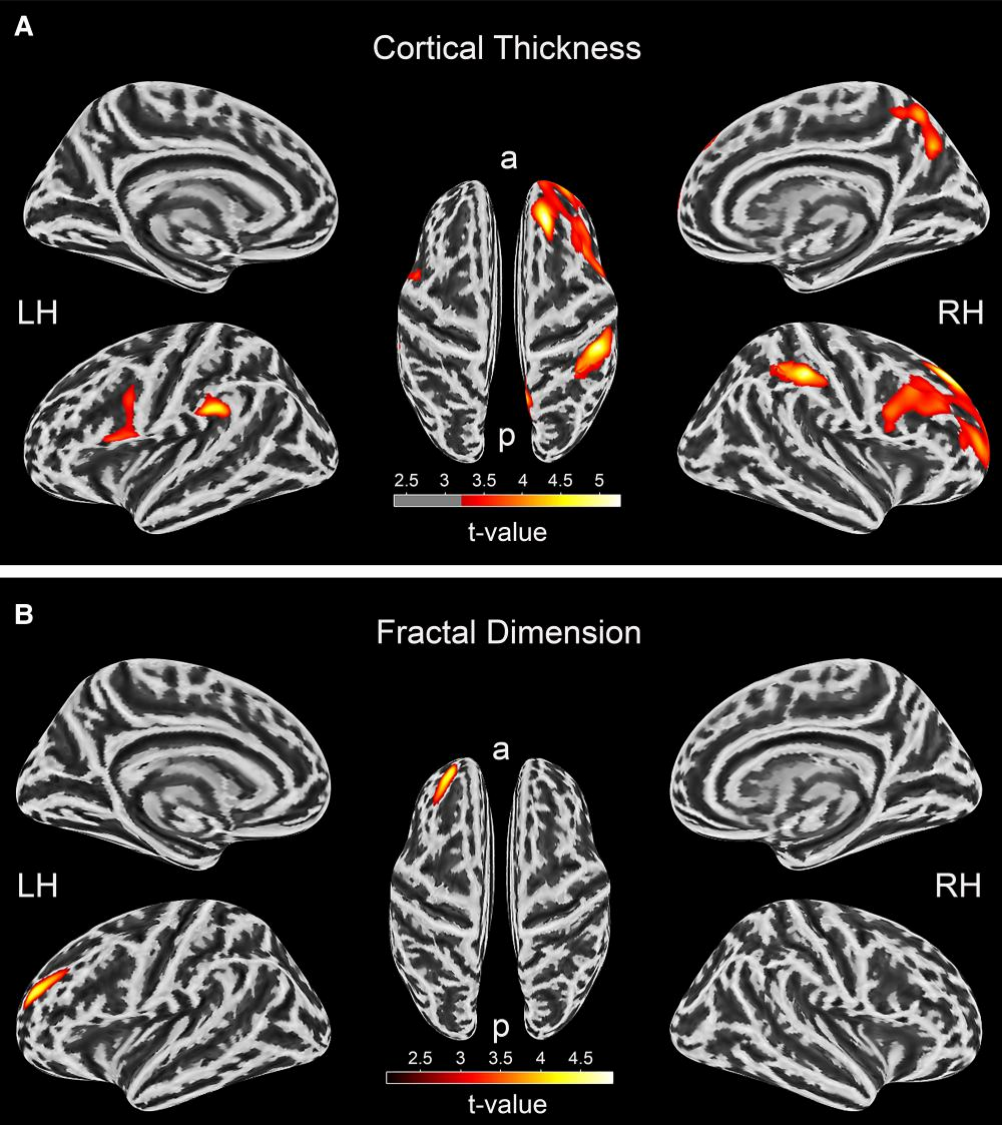
**Tumour SBM changes: (A) cortical thickness. (B) Fractal Dimension.** Two sample *t*-test; *P* < 0.05, FWE corrected, *N* = 78. T-values at vertices where the tumour group showed higher cortical thickness (**A**) and fractal dimension (**B**) compared to the normal control group are visually represented in the figure. The gradient reflects the strength of this difference, intensifying from lower to higher values. a = anterior, P = posterior, LH = left hemisphere, RH = right hemisphere

The VS group showed cortical thickening (with Cohen *d’s* small to large effect ranging from *d* = 0.80 to *d* = 1.2) in the right superior frontal gyrus (SFG), right postcentral sulcus, left supramarginal gyrus (SMG), right precuneus and left opercular part of the inferior frontal gyrus (*P_FWE_* < 0.05 at the cluster level; Table 3 and Fig. 2A).

**Table 3:** Tumour changes SBM result: *VS > nC (P < 0.05, FWE corrected)*

*P*-Value	*t*-value	Cluster Size	MNI Coordinate (mm)			Anatomical regions
			*x*	*y*	*z*	
Cortical thickness						
1.00E-06	5.30	1437	20	37	43	Right superior frontal gyrus (F1)
1.40E-05	5.20	624	42	−32	40	Right postcentral sulcus
2.20E-02	4.70	192	−56	−36	28	Left supramarginal gyrus
1.00E-03	4.30	331	9	−69	35	Right precuneus (medial part of P1)
5.00E-03	3.90	258	−53	13	7	Left opercular part of the inferior frontal gyrus
Fractal Dimensions (i.e. cortical complexity)						
2.00E-05	4.46	249	−24	44	21	Left middle frontal sulcus
			−4	−36	58	Left middle frontal gyrus (F2)
			6	−51	27	Left superior frontal sulcus

Furthermore, FDi was increased on the left hemisphere at the middle frontal sulcus (MFS), MFG and the superior frontal sulcus (SFS) in the VS group compared to controls [with Cohen d’s effect, *d* = 1.1 (*P_FWE_* < 0.05 at the cluster level; Table 3 and Fig. 2B)]. SD and GIx were not significantly different between the groups.

### Tumour Neuroplastic Changes and Relationship with PTA and Tumour Size

**Figure 3:**
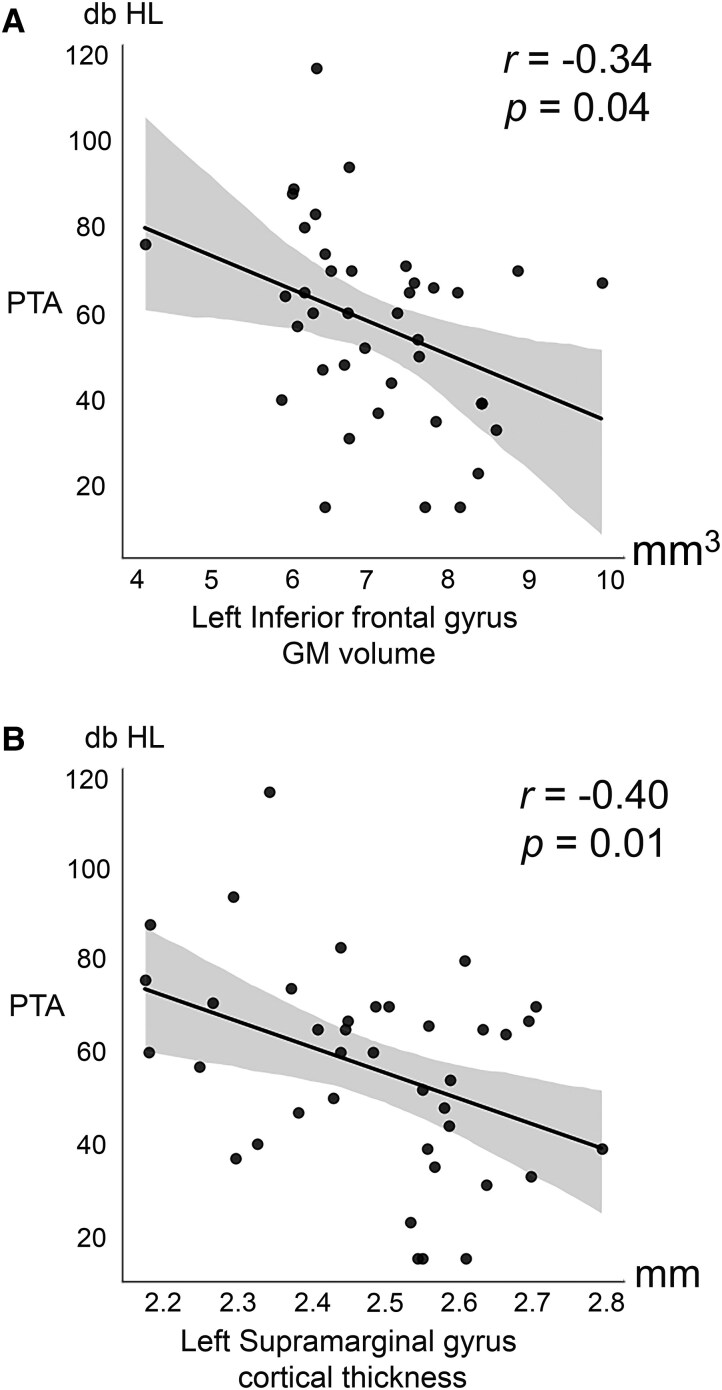
**Pure tone audiometry (PTA) correlations with the (A) grey matter (GM) volume at the left inferior frontal gyrus-triangular part and (B) cortical thickness at the left supramarginal gyrus.** db HL = decibels Hearing Level, mm= millimetres, mm^3^ = cubic millimetres. Pearson’s correlation analysis, *r*, (*N* = 40). The data points represent the imaging metrics of individual patients.

The observed GM volume increase in the left inferior frontal gyrus—triangular part was negatively correlated to PTA (*r* = −0.34, *P* = 0.04; Table 4 and Fig. 3A). Cortical thickening at the left SMG (*r* = −0.40, *P* = 0.01) was also negatively correlated to PTA (Table 4 and Fig. 3B). The regions with altered FDi had no significant relationship with PTA.

**Table 4:** Correlation between PTA and VS-associated GM volume and CT alterations

Regions	Anatomical descriptions	*r*-Value	*P-*value
*GM Volume*			
lMCC	Left middle cingulate & paracingulate gyri	−0.29	0.08
rMCC	Right middle cingulate & paracingulate gyri	−0.27	0.15
lSMA	Left supplementary motor area	−0.24	0.14
lPCL	Left paracentral lobule	−0.00	0.99
lPCUN	Left precuneus	−0.07	0.66
rSFGmedial	Right superior frontal gyrus-medial	−0.25	0.12
rSFG	Right superior frontal gyrus-dorsolateral	−0.26	0.12
lSFGmedial	Left superior frontal gyrus-medial	−0.22	0.21
rPFCventmed	Right superior frontal gyrus-medial orbital	−0.24	0.21
lMFG	Left middle frontal gyrus	−0.22	0.19
lIFGtriang	Left inferior frontal gyrus-triangular part	−0.34	0.04*
lSFG	Left superior frontal gyrus-dorsolateral	−0.23	0.16
*CT*			
rG_front_sup	Right superior frontal gyrus (F1)	−0.27	0.09
rS_postcentral	Right postcentral sulcus	−0.22	0.18
lG_pariet_inf-Supramar	Left supramarginal gyrus	−0.40	0.01*
rG_precuneus	Right precuneus (medial part of P1)	−0.07	0.66
lG_front_inf-Opercular	Left opercular part of the inferior frontal gyrus	−0.28	0.08

^*^Statistical significance*, P < 0.05*.

**Figure 4:**
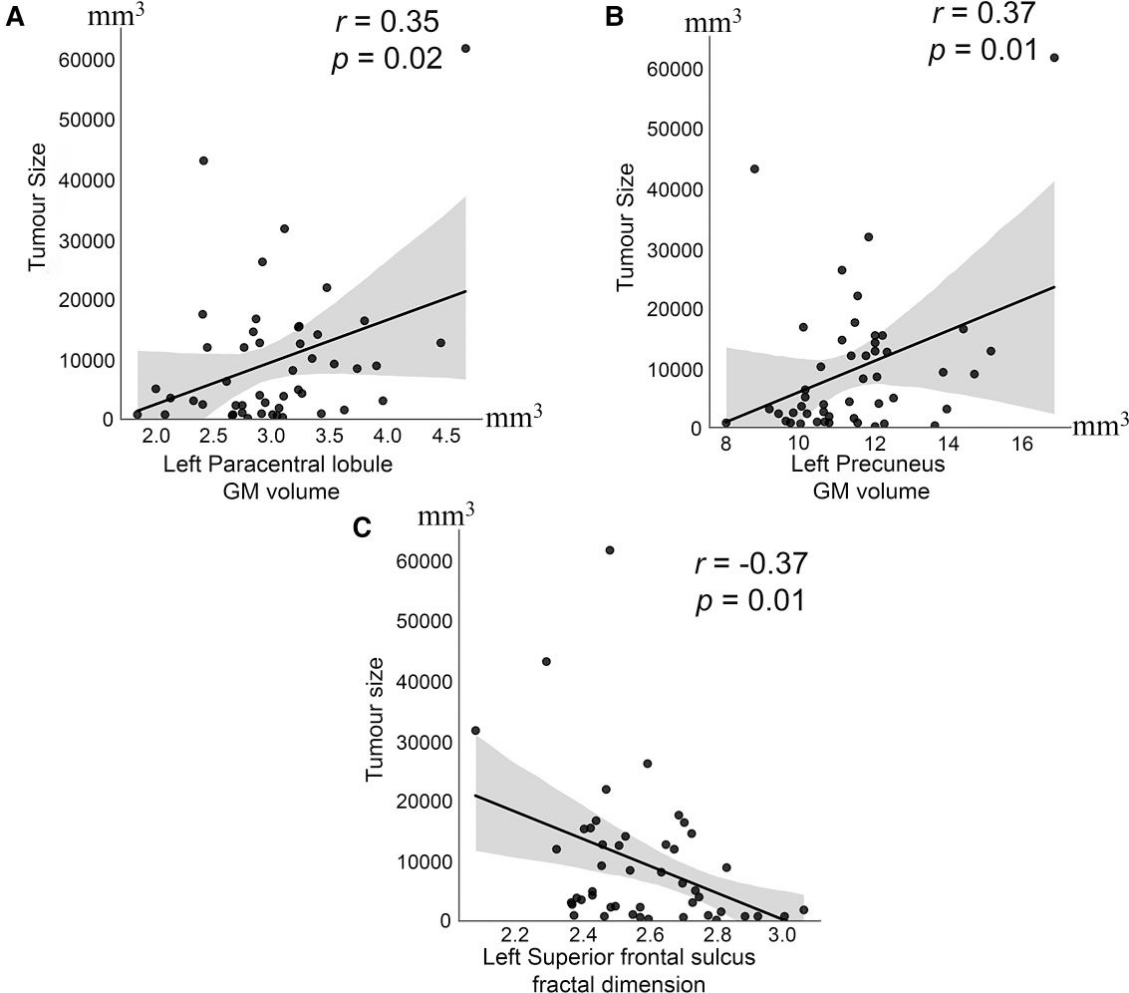
**Tumour size correlations with the (A) grey matter (GM) volume at the left paracentral lobule, (B) the left precuneus, and (C) fractal dimension at the left superior frontal sulcus.** Pearson’s correlation analysis, *r* (*N* = 48). The data points represent the imaging metrics of individual patients.

Furthermore, tumour size showed significant positive correlations with GM volume increases at the left PCL (*r* = 0.35, *P* = 0. 02; Table 5 and  Fig. 4A) and the left precuneus (*r* = 0.37, *P* = 0.01; Table 5 and Fig. 4B). The observed cortical thickening, however, had no relationship with the tumour size. In contrast, cortical complexity, captured as FDi at the left SFS (*r* = −0.37, *P* = 0.01) correlated negatively with tumour size (Table 5 and Fig. 4C).

**Table 5:** Correlation between tumour size and VS-associated GM volume and FDi alterations

Regions	Anatomical Descriptions	*r*-Value	*P-*value
*GM Volume*			
lMCC	Left middle cingulate & paracingulate gyri	0.23	0.11
rMCC	Right middle cingulate & paracingulate gyri	0.19	0.20
lSMA	Left supplementary motor area	0.16	0.28
lPCL	Left paracentral lobule	0.35	0.02*
lPCUN	Left precuneus	0.37	0.01*
rSFGmedial	Right superior frontal gyrus-medial	0.18	0.21
rSFG	Right superior frontal gyrus-dorsolateral	0.23	0.11
lSFGmedial	Left superior frontal gyrus-medial	0.15	0.32
rPFCventmed	Right superior frontal gyrus-medial orbital	0.23	0.12
lMFG	Left middle frontal gyrus	0.21	0.15
lIFGtriang	Left inferior frontal gyrus-triangular part	0.25	0.08
lSFG	Left Superior frontal gyrus-dorsolateral	0.28	0.06
*Fractal Dimension*			
lS_front_middle	Left middle frontal sulcus	0.00	0.98
lG_front_middle	Left middle frontal gyrus (F2)	−0.05	0.74
lS_front_sup	Left superior frontal sulcus	−0.37	0.01*

^*^Statistical significance, *P* < 0.05.

## Discussion

A complex interplay exists between extra-axial tumours, such as VS and cortical reorganizations. This study brought together VS tumour patients and implemented objective whole-brain morphometric techniques, including voxel- and surface-based approaches. Our findings demonstrate the ubiquitous impact of tumours on both GM volume, CT and fractal dimension.

### Tumour-related neuroplastic alterations

Morphometric changes due to VS, in addition to compression of brain tissues, also share contributions from both tinnitus and hearing loss. A recent study reported a significant increase in the CT of the precuneus, SFG, PCL, precentral gyrus, and superior parietal lobule. Some regions also demonstrated CT reduction in the VS group compared to normal controls. They, however, did not control for hearing loss or tinnitus in their analysis, so some of these reported changes might be attributable to these conditions.^9^ This was mitigated in our study by modelling covariates, and a significant increase in CT in the precuneus and SFG was replicated.^9^ We additionally found cortical thickening in the SMG, opercular part of the inferior frontal gyrus (IFG) and postcentral sulcus.

Upon evaluating the GM volume, significant increases at the precuneus and the SFG remained consistent with the CT changes and the literature, providing further evidence to support their involvement in VS pathology.^9^ The IFG, a region largely associated with language processing and executive functions also demonstrated an increase in GM volume, consistent with its observed cortical thickening in this study, suggesting a compensatory mechanism to maintain communication and function despite sensory deficits. We also found an inverse relationship of IFG with hearing loss, in line with prior studies.^28^ GM volume increases were further observed at the MCC, MFG, SMA and PCL. Interestingly, the GM volume of the left precuneus and the left PCL were positively correlated with tumour size, supporting our suggestion of neuroplastic adaptations in response to tumour compression. Furthermore, to our knowledge, this study is the first to report FDi alterations in the left MFG, MFS and SFS in VS patients.

These altered regions above are known majorly for cognitive and motor functions. Deng *et al*.,^29^ in line with our findings, reported a positive correlation between the functional connectivity of the MFG and cognitive functions and concluded that VS patients experience cognitive dysfunctions. Our results, in agreement, indicate that the observed increase in GM volume and FDi at the MFG could be an effort to compensate for the cognitive decline and other affiliated cerebellar dysfunctions. The MFS and SFS FDi increases could also be attributable to neuroplasticity to maintain cognitive and executive functions in the presence of the tumour. Moreover, a negative correlation was observed between the FDi at the SFS and tumour size; this might potentially indicate that smaller tumours allow for more cortical remodelling and functional preservation. This is a reasonable inference since smaller tumours typically do not exhibit mechanical compression of brain tissues and have little or no oedema. However, these inferences are preliminary, and more evidence is needed to understand the significance of the observed FDi increases.

Additionally, the SFG is involved in higher cognitive functions, including working memory, executive function, motor control and self-awareness. The left SFG in particular has been implicated in spatially oriented processing.^30^ Our observed structural increases could indicate an adaptive response to increased cognitive demands required to compensate for vestibular deficits. The SFG's involvement in multisensory integration further underscores its robustness in our analysis.

Interestingly, studies using different metrics and modalities corroborate our findings of significant alterations in the non-auditory frontal regions in VS patients. A recent positron emission tomography (PET) study showed increased expression of translocator proteins (TSPO) following microglial activation in brain regions later identified by voxel-based cluster analysis to include non-auditory motor and premotor frontal regions in VS patients with growing tumours. They inferred that these findings, although suggestive of neuroinflammation, could also reflect structural reorganizations.^31^ In a similar vein, Deng *et al*.,^32^ using tract-based spatial analysis, compared diffusion tensor imaging (DTI) metrics and reported higher fractional anisotropy (FA) in the left superior longitudinal fasciculus and bilateral corticospinal tract of VS patients, which was attributed to increased axial diffusivity (AD) in these regions, most likely due to the destruction of the integrity of axons. They further found decreased FA in the forceps minor, another significant fibre bundle in the frontal region.^32^ Although preliminary, our findings, together with these existing studies, highlight the significant involvement of the frontal region in VS pathology, which has been implicated in the cognitive decline observed in these patients.^29^ Current evidence suggests that both neuroinflammation and cortical reorganization may drive the observed alterations. Alternatively, inflammation might underlie these reorganizations in corresponding regions, helping to maintain function. Further tailored research is, however, needed to elucidate the specific mechanisms behind these findings.

The precuneus is one of the most connected hubs in the human cerebrum. On a fine-resolution network map, it has subregions associated with three different brain networks: the default mode network (DMN) core, the DMN episodic memory subnetwork, and the para-cingulate network (PCgN).^33^ Given the extensive connection of the precuneus, damage to it has been established to trigger various morphological cascades relating to the underlying pathology.^34^ Function-wise, it plays a role in self-awareness, visuospatial processing, episodic memory retrieval and some aspects of consciousness. VS originating from the auditory and vestibular nerve, often results in sensory deafferentation leading to functional impairment such as spatial disorientation.^35^ The observed increase in GM volume and CT could represent efforts to mitigate this disruption.

Finally, we found alterations in the left supramarginal gyrus—a region responsible for spatial attention and sensorimotor integration. The volume of the SMG has also been associated with the ability to recognize emotions.^36^ Given that the cerebellum has a similar function already described by previous studies,^37^ it can be suggested that our observed SMG cortical thickening is an effort to ‘make up’ potentially cerebellar compression-induced functional deficits (as related to motor coordination and certain cognitive functions). Our results further indicate a thinner left SMG cortex in hearing loss patients, which could be atrophy of disuse given its involvement in auditory memory.^38^

### Proposed para-cingulate network alterations in vestibular schwannoma

The PCgN located in the precuneus has parcellations across the superior parietal cortex, posterior cingulate cortex (PCC), the PCL and MCC, and the anterior cingulate and medial prefrontal cortex. Our discovery of cortical thickening in the precuneus and GM volume increase in the precuneus, MCC and PCL demonstrate a significant involvement of this network in VS-related neuroplasticity. Relating structures to functions, the PCgN has shown functional connectivity (FC) across several regions encompassing the MFG, PCL, pre-/supplementary motor area, pre/postcentral gyrus, SFG, etc.^33,39^ This network's functions include self-knowledge, sensorimotor dynamics, planning, external agency, working memory, etc. To our knowledge, ours is the first study to report the potentially significant involvement of VS-related neuroplastic changes with the recently described PCgN. Further understanding of the mechanisms involved may potentiate novel targets for neuromodulation in VS management, which will contribute to an overall improvement of patients’ quality of life.

### Clinical applications and Prospects

With advancements in technology and improvements in clinical skills, the need for precise patient care has become increasingly critical.^40^ While our findings in this study are preliminary, they may have significant implications in various management decision-making scenarios, such as facilitating more targeted rehabilitation for VS patients’ pre- and/or post-surgical intervention. Understanding the regions altered by the tumour, distinct from those affected by tinnitus, hearing loss, or other symptoms, could enable more individualized symptomatic care. Furthermore, these findings could contribute to developing pharmacological agents or refining neurostimulation techniques^41^ to enhance neuroplasticity in specific brain regions for therapeutic purposes.

Additionally, neurofeedback therapy using fMRI has shown promise in alleviating tinnitus distress.^42^ This has been made possible by identifying structural and functional changes in the brain associated with tinnitus. Further research into the specific alterations and their causes following VS could pave the way for this non-invasive therapy to improve the quality of life of patients, especially those with surgical contraindications or those who prefer not to undergo surgical resection.

### Study Limitations

The retrospective nature of our study exposes it to several limitations. We only utilized preoperative data in our analysis and did not consider the effects of treatments on brain structures. Also, we only used structural MRI data; more multimodal information would provide a more nuanced understanding of how the brain adapts to VS. Furthermore, we utilized MRI data from multiple machines. This remains an issue despite bias corrections and harmonization and our study should be interpreted with that in mind. Additionally, the effects of affective disorders such as anxiety and depression, which are often comorbid with VS, were not considered in this study and are another limitation to consider, as these have also been reported to cause morphometric alterations. Finally, given the lack of longitudinal data, this study did not consider tumour growth rate, which has also been implicated in morphometric alterations.

## Conclusion

This study provides strong evidence to support the significant involvement of the precuneus, superior frontal gyrus and inferior frontal gyrus in VS. It further reinforces the critical role of tumour size in VS management, emphasising its importance as a key parameter in clinical decision-making. Our findings also underscore the necessity of incorporating tumour size into the development of more accurate analytical models for future research, ultimately contributing to the advancement of personalized and effective care for patients. Finally, our results tentatively suggest the involvement of other factors besides hearing loss and tinnitus in VS neuroplasticity, a better understanding of which might bring about novel therapy for VS, especially non-surgical symptomatic treatment options.

## Supplementary Material

fcaf107_Supplementary_Data

## Data Availability

Due to privacy concerns, the data supporting our findings cannot be shared publicly. However, access may be granted to researchers who meet ethical criteria through the Ethics Review Committee of Xiangya Hospital, Central South University (email: xyyyllwyh@126.com).
