# Supplementary material for: Brain structural alterations in vestibular schwannoma beyond tinnitus and hearing loss
Source: Brain Commun. 2025 Mar 11;7(2):fcaf107. doi: 10.1093/braincomms/fcaf107 (PMC11937892; doi:10.1093/braincomms/fcaf107)
Supplement: fcaf107_Supplementary_Data [file fcaf107_supplementary_data.docx]

Supplementary Material

| Table 1 MRI parameters | | | | | | | | | |  |
| --- | --- | --- | --- | --- | --- | --- | --- | --- | --- | --- |
| Site | Manufacturer | Model Name | Magnetic Field Strength | Echo Time | Repetition Time | Slice Thickness | Flip Angle | N subjects | Age range | Groups |
| Xiangya Hospital | Siemens | Aera | 1.500 | 0.004 | 1.722 | 1.200 | 8.000 | 3.000 | [35,51] | Patients |
| Fei Yu hospital | GE | DISCOVERY MR750w | 3.000 | 0.003 | 0.009 | 1.000 | 12.000 | 4.000 | [34,51] | Patients |
| Xiangya Hospital | Siemens | Prisma | 3.000 | 0.003 | 2.027 | 1.000 | 8.000 | 15.000 | [17,59] | Patients & Controls |
| Xiangya Hospital | GE | SignaArchitect | 3.000 | 0.003 | 0.007 | 1.000 | 12.000 | 8.000 | [29,74] | Patients |
| Xiangya Hospital | GE | SignaHDxt | 3.000 | 0.002 | 0.006 | 1.600 | 15.000 | 20.000 | [20,72] | Patients |
| Xiangya Hospital | GE | SignaPremier | 3.000 | 0.003 | 0.597 | 1.000 | 12.000 | 4.000 | [15,68] | Patients |
| Xiangya Boai Hospital | Siemens | Skyra | 3.000 | 0.003 | 5.000 | 1.000 | 8.000 | 24.000 | [21,58] | Controls |

**Supplementary Table 1** MRI Parameters


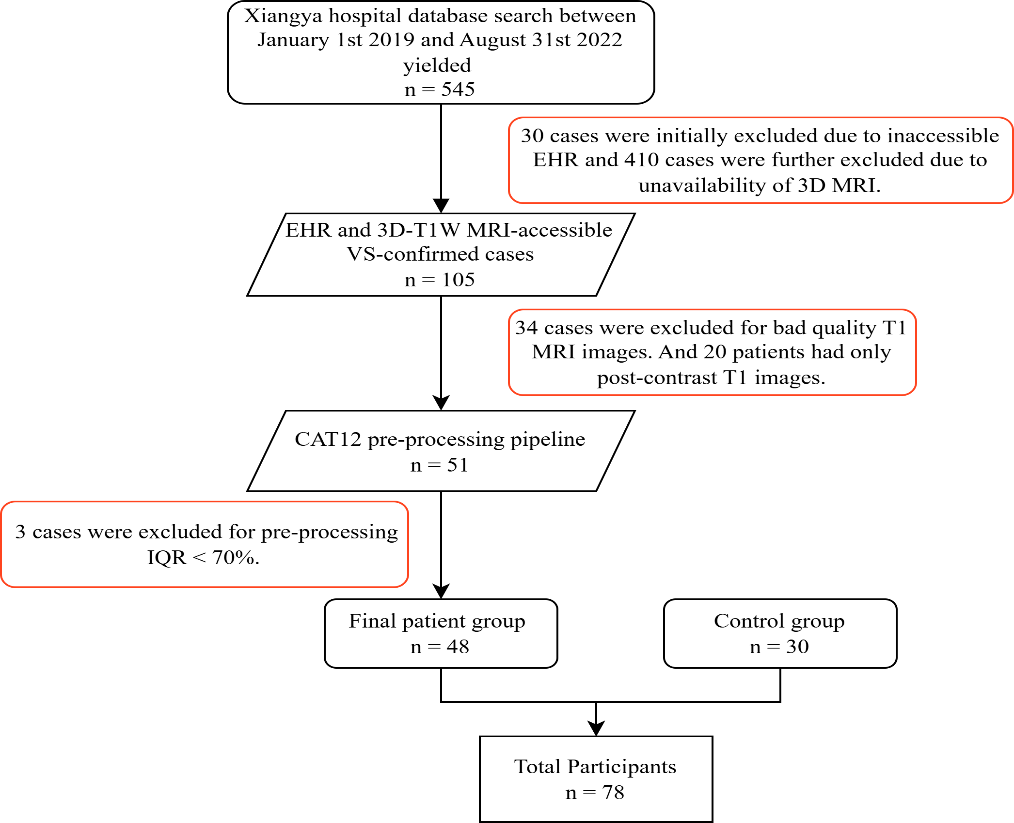


**Supplementary Figure 1. A flowchart of the various inclusion and exclusion stages. (**EHR = Electronic Health Record, 3D-T1W = 3-dimensional T1-weighted, MRI = Magnetic Resonance Imaging, VS = Vestibular Schwannoma, CAT12 = Computational Anatomy Toolbox 12, IQR = Image Quality Rating, n = number of participants.)
